# Development and Validation of a Score to Predict Acute Care Use After Initiation of Systemic Therapy for Cancer

**DOI:** 10.1001/jamanetworkopen.2019.12823

**Published:** 2019-10-09

**Authors:** Robert C. Grant, Rahim Moineddin, Zhan Yao, Melanie Powis, Vishal Kukreti, Monika K. Krzyzanowska

**Affiliations:** 1 Division of Medical Oncology and Hematology, Princess Margaret Cancer Centre, University Health Network, Toronto, Ontario, Canada; 2 Department of Medicine, University of Toronto, Toronto, Ontario, Canada; 3 ICES, Toronto, Ontario, Canada

## Abstract

**Importance:**

Emergency department visits and hospitalizations after starting systemic therapy for cancer are frequent, undesirable, and costly. A score to quantify the risk of needing acute care can inform decision-making and facilitate the development of preventive interventions.

**Objective:**

To develop and validate a score to predict early use of acute care after initiating systemic therapy for cancer.

**Design, Setting, and Participants:**

A retrospective population-based cohort study was conducted between July 1, 2014, and June 30, 2015. Patients with cancer were eligible if they started a new systemic therapy for cancer, regardless of line of therapy. A total of 12 162 patients in Southwestern Ontario, Canada, formed the development cohort and 15 845 patients in Northeastern Ontario formed the validation cohort. Data analysis was conducted from December 1, 2016, to August 10, 2019.

**Exposures:**

The Prediction of Acute Care Use During Cancer Treatment (PROACCT) score was created based on logistic regression in the development cohort. Combinations of cancer type and regimens were grouped into quintiles based on risk of needing acute care. The score was assessed in the validation cohort.

**Main Outcomes and Measures:**

At least 1 emergency department visit or hospitalization within 30 days after starting systemic therapy for cancer identified from administrative databases.

**Results:**

Among the 12 162 patients in the development cohort, 6903 were women and 5259 were men (mean [SD] age, 62.9 [12.6] years); among the 15 845 patients in the validation cohort, 9025 were women and 6820 were men (mean [SD] age, 62.9 [12.6] years). Use of acute care occurred within 30 days after initiation of systemic therapy in 3039 patients (25.0%) in the development cohort and 4212 patients (26.6%) in the validation cohort. Three characteristics predicted early use of acute care and formed the PROACCT score: combination of cancer type and treatment regimen, age, and emergency department visits in the prior year (C statistic, 0.67; 95% CI, 0.66-0.69; *P* < .001). Other characteristics including patient-reported symptoms did not improve performance. In the validation cohort, the PROACCT score was associated with use of acute care (odds ratio per point increase, 1.22; 95% CI, 1.20-1.24; *P* < .001), had a C statistic of 0.61 (95% CI, 0.60-0.62; *P* < .001), was reasonably calibrated, and provided net benefit in decision curve analysis.

**Conclusions and Relevance:**

The PROACCT score predicted the risk of early use of acute care in patients starting systemic treatment for cancer and could be incorporated at the point of care to select patients for preventive interventions. Future studies should validate the PROACCT score in other settings.

## Key Points

**Question:**

Is it possible to predict which patients with cancer will require early acute care after starting systemic therapy?

**Findings:**

In a cohort study, using population-based administrative databases in Ontario, Canada, a score was developed and validated to predict a visit to the emergency department or a hospitalization within the first 30 days after starting systemic therapy for cancer based on cancer type, systemic therapy regimen, previous emergency department visits, and age.

**Meaning:**

The findings suggest that patients at high risk for use of acute care after starting systemic therapy for cancer can be identified using a simple score that is available at the point of care.

## Teaser

This cohort study uses population-based administrative and clinical databases to develop and validate a score to predict early use of acute care after initiating systemic therapy for cancer.

## Introduction

Use of acute care is common during systemic therapy for cancer. A systematic review estimated that almost half of patients in observational studies are hospitalized while receiving chemotherapy.^1^ Use of acute care is undesirable because it may reflect suboptimal control of disease-related symptoms or toxic effects of treatment and constitutes the largest component of cancer care costs.^2^ The risk of acute care use with cancer treatment should be included as part of the informed consent process because this information can influence treatment decisions.^3^ Some acute care use is preventable^4^ and interventions can reduce acute care use, morbidity, and costs.^5,6^ Reducing acute care use is a strategic priority of health care policy makers including Cancer Care Ontario^7^ (CCO) and the Center for Medicare and Medicaid Innovation.^8^ In Ontario specifically, CCO plans to develop a “standardized and comprehensive approach to managing treatment-related toxicity,”^7^ including “a standardized process/tool for stratifying patients by toxicity risk.”^9^

Strategies to identify patients at risk for acute care use can facilitate research on prevention. Although several existing risk scores predict specific toxic effects from chemotherapy such as febrile neutropenia,^10,11,12^ prediction scores for acute care use have focused only on hospitalizations after starting palliative chemotherapy for solid tumors and have not used population-based data.^13,14^ To our knowledge, to date, population-based studies on acute care use during systemic treatment for cancer have focused mostly on a single cancer type or considered few risk factors and have not generated a predictive model.^15,16,17,18,19^

In this study, we developed and validated the Prediction of Acute Care Use During Cancer Treatment (PROACCT) score to estimate the probability of acute care use within 30 days after the initiation of systemic therapy for solid and hematologic cancers using population-based administrative data. Thirty days was selected because most acute care use after initiating systemic therapy for cancer occurs during this period^20^ and because early use of acute care may be the most relevant to the design and evaluation of preventive interventions.

## Methods

### Cohort Creation

The cohort consisted of patients in Ontario who initiated an anticancer systemic therapy during the accrual period between July 1, 2014, and June 30, 2015. The cohort was created by linking multiple population-based administrative and clinical databases. These databases were linked using unique, encoded identifiers and analyzed at the Institute for Clinical Evaluative Sciences. Health care professionals and hospitals in Ontario are reimbursed for services contingent on the provision of administrative data. As a result, administrative databases in Ontario capture more than 95% of health care services rendered.^21^ This study was conducted in accordance with the Transparent Reporting of a Multivariable Prediction Model for Individual Prognosis or Diagnosis (TRIPOD) statement.^22^ The Sunnybrook Research Institute Research Ethics Board approved this study. Analyses were undertaken at the Institute for Clinical Evaluative Sciences, a prescribed entity under Ontario’s Personal Health Information Privacy Act. As such, individual consent is not required for the use of data for approved research projects.

Patients were identified using the Activity Level Reporting database maintained by CCO.^23^ Patients were excluded if they were treated for a rare cancer (defined as <20 patients in the development cohort), a myeloproliferative neoplasm, or cancers commonly treated on an inpatient basis, such as acute leukemias. Patients were also excluded if their systemic therapy was started on an inpatient basis, they were concurrently being treated for multiple cancers, they were younger than 18 years, they did not have a valid Ontario Health Insurance number, or they had a missing value for age, sex, or region of residence.

Systemic therapy treatment courses were included if they were initiated during the accrual period. Initiation of a new treatment regimen was defined as the first dose of a systemic therapy within the accrual period, with no evidence of the same regimen in the prior 90 days. Only the first new regimen within the accrual period for each patient was included. Regimens were included if they were a funded, evidence-based regimen for the patient’s primary cancer and indication (ie, curative or palliative) according to CCO’s Systemic Treatment Funding Model. The Systemic Treatment Funding Model was developed in consultation with clinical experts and administrators, who systematically reviewed the literature to identify which treatment regimens have evidence of efficacy for each cancer type and indication.^24,25,26^ Cancer centers in Ontario are reimbursed for the provision of systemic therapy only if the therapy is approved within the Systemic Treatment Funding Model. We excluded regimens outside of the Systemic Treatment Funding Model because these regimens should not be used in Ontario and are not reliably captured. More than 94% of systemic therapy treatments delivered in Ontario follow the CCO Systemic Treatment Funding Model.^24^ Systemic therapies included intravenous and oral cytotoxic or targeted agents. Supportive regimens such as bisphosphonates and most hormonal agents were excluded, with the exception of second-line hormonal agents for prostate cancer. Regimens received by fewer than 10 patients in the development cohort were excluded.

### Outcomes

The primary outcome was the occurrence of at least 1 emergency department (ED) visit or hospitalization during the first 30 days after the initiation of systemic therapy (AC30), regardless of the reason or duration. Emergency department visits were ascertained from the National Ambulatory Care Reporting System database^27^ and hospitalizations from the Canadian Institute for Health Information Discharge Abstract Database.^28^

### Baseline Characteristics

We generated a list of characteristics that had prior evidence for an association with acute care use based on a systematic review of the literature^29^ and data availability. Age, sex, cancer diagnosis, systemic therapy regimen, radiotherapy treatments, and facility level where care was received were identified from the Activity Level Reporting database. Cancer diagnosis was identified using *International Classification of Diseases* codes, which were matched to diagnostic groupings in the CCO Systemic Treatment Funding Model. Facility level ranged from 1 through 4, where 1 represents centers with investigational new drug programs, 2 represents centers that perform high-complexity procedures such as head and neck chemoradiotherapy, 3 represents centers with medical oncologists on site, and 4 represents satellite centers without medical oncologists on site.^30^ Postal code from the Ontario Registered Persons Database classified residence by income quintile and as rural or urban.^31^ Comorbidities were assessed using Aggregated Diagnosis Groups (The Johns Hopkins ACG System, Version 10),^32^ which incorporate information from multiple databases. Symptoms were self-reported using the Edmonton Symptom Assessment System (ESAS); scores were dichotomized, where a score of 4 or greater reflected moderate or severe symptoms.^33,34^ Performance status was self-reported using the Eastern Cooperative Oncology Group (ECOG) scale. The ESAS and ECOG scores came from the Symptom Management and Reporting Database.^35,36^ The ESAS and ECOG values included were those closest to the regimen start date, as long as they were recorded within the preceding 30 days. Emergency department visits and hospitalizations within the previous year were identified using the same approach as with AC30.

### Statistical Analysis

#### Risk Score Development and Validation

Ontario is split into 14 geographic regions called Local Health Integration Networks for health care delivery. Patients from Southwestern Ontario formed the development cohort (Local Health Integration Networks 1-6); patients from Central, Eastern, and Northern Ontario formed the validation cohort (Local Health Integration Networks 7-14). Cancer type and regimen (ie, colorectal, FOLFIRI [leucovorin, fluorouracil, and irinotecan] plus bevacizumab) were combined into a single characteristic and sorted into quintiles based on the observed mean number of ED visits or hospitalizations within the first 30 days after starting treatment in the development cohort (eTable in the Supplement). We combined cancer type and regimen into a single characteristic to avoid statistical issues of collinearity and complete separation.

The risk score was based on a multivariable logistic regression estimated in the development cohort with AC30 as the outcome using a backward selection procedure (2-tailed *P* < .05 was considered significant). Aggregated Diagnosis Groups, income quintile, rural residence, facility level, the receipt of palliative radiotherapy, ESAS score, and ECOG score were excluded from the selection procedure because these characteristics may not be readily available to clinicians and had a limited association with score performance in a sensitivity analysis. Treatment courses were excluded if there was a missing value for a characteristic included in the regression (complete case analysis). Coefficients from the multivariable regression model were converted into integer “points” to create the score.^37^ The points for each characteristic are summed to generate an individualized score.

The risk score was assessed in the validation cohort. The predicted probability of AC30 came from a logistic regression using the score from the development cohort. Discrimination was evaluated visually with the receiver operating characteristic curve and statistically using the C statistic.^38^ Calibration was evaluated visually using a locally estimated scatterplot smoothing curve comparing predicted with observed AC30 proportions.^38^ Statistical testing for calibration was not performed because the large sample size could make small departures in the observed from predicted values seem significant.^39^ Decision curve analysis assessed the potential clinical benefits of using the PROACCT score to select patients for an intervention to prevent AC30. Decision curve analysis is described in detail elsewhere.^40^ In brief, decision curve analysis evaluates the value of a predictive model when making a clinical decision, for instance, selecting patients for a preventive intervention. Three strategies are compared: selecting all patients for the intervention (treat all), selecting no patients (treat none), and selecting patients using the predictive model. In decision curves, the x-axis depicts the threshold probability, which is chosen by the decision-maker. In the context of this study, a decision-maker would compare the strategies at a threshold probability of 0.2 if he or she was willing to apply the intervention to 5 patients if one of them would otherwise have had an AC30 with certainty.^41^ The threshold probability selected by a decision-maker will depend on the relative costs and benefits of the intervention, which can include health, financial, and other considerations, so a range is provided. The y-axis depicts the net benefit of each strategy, which is expressed in terms of the value of true positives. For example, a net benefit of 0.1 is the value of applying the preventive intervention to 10% of patients, all of whom would have had an AC30 with certainty otherwise. Data analysis was conducted from December 1, 2016, to August 10, 2019. Analyses were performed in SAS, version 7.1 (SAS Institute Inc).

#### Sensitivity Analysis

We evaluated whether the addition of facility level, Aggregated Diagnosis Groups, income quintile, ESAS score, ECOG score, and receipt of radiotherapy improved the model. We assessed how the score performed at predicting each individual component of AC30 separately: ED visits that did not lead to hospitalizations, and hospitalizations. We also assessed the score in the subgroups of patients treated with palliative and nonpalliative intent.

## Results

### Cohort Description

We identified 12 162 patients with treatment initiations in the development cohort and 15 845 patients with treatment initiations in the validation cohort (Table 1). Among the 12 162 patients in the development cohort, 6903 were women and 5259 were men (mean [SD] age, 62.9 [12.6] years); among the 15 845 patients in the validation cohort, 9025 were women and 6820 were men (mean [SD] age, 62.9 [12.6] years). Patient and treatment characteristics were similar in both cohorts, except that more patients in the validation cohort lived in rural settings and were treated in centers without the capacity for investigational drug programs or high-complexity procedures.

**Table 1.** Baseline Characteristics of Patients Starting Treatment

Characteristic	Patients, No. (%)	
	Development Cohort (n = 12 162)	Validation Cohort (n = 15 845)
Female	6903 (56.8)	9025 (57.0)
Age, y		
18-44	978 (8.0)	1257 (7.9)
45-64	5336 (43.9)	6852 (43.2)
65-74	3623 (29.8)	4763 (30.1)
>74	2225 (18.3)	2973 (18.8)
Rural residence	1355 (11.1)	2648 (16.7)
Income quintile		
1	1900 (15.6)	2951 (18.6)
2	2296 (18.9)	3091 (19.5)
3	2607 (21.4)	2946 (18.6)
4	2675 (22.0)	3402 (21.5)
5	2652 (21.8)	3389 (21.4)
Missing	32 (0.3)	66 (0.4)
Aggregated diagnosis groups		
0-5	1324 (10.9)	1718 (10.8)
6-10	4915 (40.4)	6518 (41.1)
>10	5923 (48.7)	7609 (48.0)
ED visit in prior year	7090 (58.3)	9440 (59.6)
Hospitalization in prior year	5631 (46.3)	7560 (47.7)
Cancer type^a^		
Gastrointestinal	2912 (23.9)	3924 (24.8)
Breast	2586 (21.3)	3554 (22.4)
Hematologic	1987 (16.3)	2498 (15.8)
Lung	1644 (13.5)	2169 (13.7)
Genitourinary	955 (7.9)	1219 (7.7)
Gynecological	880 (7.2)	1155 (7.3)
Other	1198 (9.9)	1326 (8.4)
ESAS score (4 or higher)		
Pain	1942 (16.0)	2620 (16.5)
Missing	4242 (34.9)	4795 (30.3)
Tiredness	3296 (27.1)	4300 (27.1)
Missing	4240 (34.9)	4789 (30.2)
Drowsiness	1904 (15.7)	2463 (15.5)
Missing	4243 (34.9)	4788 (30.2)
Nausea	633 (5.2)	882 (5.6)
Missing	4243 (34.9)	4791 (30.2)
Lack of appetite	1879 (15.4)	2456 (15.5)
Missing	4243 (34.9)	4794 (30.3)
Shortness of breath	1454 (12.0)	2039 (12.9)
Missing	4249 (34.9)	4788 (30.2)
Depression	1630 (13.4)	2220 (14.0)
Missing	4246 (34.9)	4787 (30.2)
Anxiety	2459 (20.2)	3246 (20.5)
Missing	4246 (34.9)	4786 (30.2)
Well-being	2941 (24.2)	4129 (26.1)
Missing	4246 (34.9)	4812 (30.4)
ECOG score		
0 or 1	5649 (46.4)	7469 (47.1)
2	1203 (9.9)	1529 (9.7)
3 or 4	941 (7.7)	1101 (7.0)
Missing	4369 (35.9)	5746 (36.3)
Systemic therapy intent		
Curative, adjuvant, or neoadjuvant	5090 (41.9)	6738 (42.5)
Palliative	7072 (58.2)	9107 (57.5)
Cancer type–regimen AC30 risk, No./total No. (%)		
Very low	2675/11 853 (22.6)	3012/15 460 (19.5)
Low	3171/11 853 (26.8)	4388/15 460 (28.4)
Moderate	2677/11 853 (22.6)	3794/15 460 (24.5)
High	2002/11 853 (16.9)	2547/15 460 (16.5)
Very high	1328/11 853 (11.2)	1719/15 460 (11.1)
Radiotherapy		
Curative intent		
Concurrent	1525 (12.5)	1748 (11.0)
Prior 60 d	1258 (10.3)	1626 (10.3)
Palliative intent		
Concurrent	462 (3.8)	823 (5.2)
Prior 60 d	870 (7.2)	1249 (7.9)
No radiotherapy	8047 (66.2)	10 399 (65.6)
Facility level^b^		
1	6750 (55.5)	7268 (45.9)
2	2747 (22.6)	3973 (25.1)
3	1948 (16.0)	3810 (24.0)
4	689 (5.7)	739 (4.7)
Missing	28 (0.2)	55 (0.4)

Abbreviations: AC30, at least 1 acute care use in the 30 days after starting systemic therapy; ECOG, Eastern Cooperative Oncology Group; ED, emergency department; ESAS, Edmonton Symptom Assessment System.

^a^ Detailed information on cancer type is presented along with the treatment regimens in the eTable in the Supplement.

^b^ Level 1 represents centers with investigational new drug programs, 2 represents centers that perform high-complexity procedures such as head and neck chemoradiotherapy, 3 represents centers with medical oncologists on site, and 4 represents satellite centers without medical oncologists on site.^30^

At least 1 ED visit or hospitalization during the first 30 days after the initiation of systemic therapy occurred during 3039 treatments (25.0%) in the development cohort and 4212 treatments (26.6%) in the validation cohort. Combinations of cancer type and treatment regimen with the highest rate of AC30 included cancers with poor prognoses such as pancreatic and lung cancer, and regimens with high toxic effects such as combinations including cisplatin and docetaxel (eTable in the Supplement). In contrast, indolent cancers treated with monotherapy had lower rates of AC30.

### Development of the PROACCT Score

In univariable regressions in the development cohort, AC30 was significantly associated with all characteristics except sex, rural residence, and curative-intent radiotherapy (Table 2). The C statistic of the full multivariable model in the development cohort was 0.70 (95% CI, 0.69-0.71; *P* < .001). In the simple multivariable model using backward selection excluding characteristics that might not be readily accessible at the bedside (income quintile, rural residence, radiotherapy, and facility level) or those with frequently missing values (ECOG score and ESAS score), the C statistic was 0.68 (95% CI, 0.66-0.69; *P* < .001).

**Table 2.** Univariable and Multivariable Logistic Regression Models for Emergency Department Visit or Hospitalization Within 30 Days After Starting New Course of Systemic Therapy

Characteristic	Model					
	Univariable		Full Multivariable		Final Multivariable	
	OR (95% CI)	*P* Value	OR (95% CI)	*P* Value	OR (95% CI)	*P* Value
Female vs male	0.99 (0.89-1.10)	.85	1.05 (0.93-1.19)	.39	NA	NA
Age, y						
18-44	1.15 (0.94-1.41)	.17	1.33 (1.07-1.65)	.008	1.3 (1.05-1.60)	.02
45-64	1 [Reference]	NA	1 [Reference]	NA	1 [Reference]	NA
65-74	1.13 (1.00-1.29)	.05	1.09 (0.94-1.24)	.23	1.11 (0.97-1.27)	.12
>74	1.06 (0.91-1.24)	.44	1.15 (0.97-1.37)	.10	1.22 (1.04-1.44)	.02
Income quintile						
1	1.21 (1.02-1.44)	.03	1.08 (0.89-1.30)	.44	NA	NA
2	1.00 (0.84-1.19)	.99	0.92 (0.76-1.10)	.34	NA	NA
3	1.06 (0.90-1.25)	.47	1.02 (0.85-1.21)	.84	NA	NA
4	0.94 (0.79-1.10)	.43	0.90 (0.75-1.07)	.23	NA	NA
5	1 [Reference]	NA	NA	NA	NA	NA
Rural vs urban residence	1.17 (0.98-1.40)	.09	1.23 (1.01-1.49)	.04	NA	NA
Aggregated Diagnosis Groups						
0-5	1 [Reference]	NA	1 [Reference]	NA	NA	NA
6-10	1.40 (1.13-1.72)	<.001	1.16 (0.93-1.45)	.18	NA	NA
>10	2.32 (1.89-2.86)	.002	1.63 (1.29-2.05)	<.001	NA	NA
ED visit past 12 mo	1.99 (1.78-2.23)	<.001	1.57 (1.37-1.80)	<.001	1.88 (1.68-2.12)	<.001
Hospitalization past 12 mo	1.34 (1.20-1.49)	<.001	0.93 (0.81-1.05)	.24	NA	NA
ESAS score ≥4						
Pain	1.65 (1.46-1.86)	<.001	1.04 (0.89-1.21)	.58	NA	NA
Tiredness	1.68 (1.51-1.87)	<.001	1.03 (0.87-1.21)	.71	NA	NA
Drowsiness	1.78 (1.58-2.00)	<.001	1.12 (0.94-1.32)	.18	NA	NA
Nausea	1.92 (1.60-2.30)	<.001	1.06 (0.85-1.31)	.59	NA	NA
Lack of appetite	1.89 (1.67-2.13)	<.001	1.18 (1.01-1.37)	.04	NA	NA
Shortness of breath	1.78 (1.57-2.03)	<.001	1.17 (1-1.37)	.05	NA	NA
Depression	1.65 (1.45-1.87)	<.001	0.99 (0.82-1.18)	.88	NA	NA
Anxiety	1.59 (1.42-1.78)	<.001	1.13 (0.97-1.32)	.11	NA	NA
Well-being	1.78 (1.59-1.98)	<.001	1.13 (0.97-1.32)	.11	NA	NA
ECOG score						
0 or 1	1 [Reference]	NA	1 [Reference]	NA	NA	NA
2	1.47 (1.27-1.70)	<.001	1.01 (0.85-1.20)	.87	NA	NA
3 or 4	2.10 (1.80-2.44)	<.001	1.21 (1.00-1.46)	.046	NA	NA
Nonpalliative-intent systemic treatment	0.84 (0.77-0.91)	<.001	1.09 (0.94-1.26)	.24	NA	NA
Cancer type–regimen AC30 risk						
Very low	1 [Reference]	NA	1 [Reference]	NA	1 [Reference]	NA
Low	1.81 (1.48-2.21)	<.001	2.02 (1.62-2.50)	<.001	1.96 (1.59-2.41)	<.001
Moderate	2.68 (2.19-3.28)	<.001	2.87 (2.32-3.53)	<.001	2.88 (2.34-3.53)	<.001
High	4.08 (3.34-4.97)	<.001	3.89 (3.16-4.79)	<.001	4.18 (3.41-5.12)	<.001
Very high	5.98 (4.82-7.42)	<.001	5.55 (4.42-6.95)	<.001	5.92 (4.75-7.37)	<.001
Radiotherapy						
Curative intent						
Concurrent	0.89 (0.75-1.05)	.17	0.93 (0.74-1.17)	.55	NA	NA
Prior 60 d	0.93 (0.78-1.11)	.40	1.02 (0.80-1.29)	.86	NA	NA
Palliative intent						
Concurrent	1.32 (1.02-1.70)	.03	1.20 (0.90-1.58)	.20	NA	NA
Prior 60 d	2.10 (1.80-2.44)	<.001	1.24 (1.01-1.51)	.04	NA	NA
Facility level^a^						
1	1 [Reference]	NA	NA	NA	NA	NA
2	1.08 (0.96-1.22)	.20	1.10 (0.97-1.25)	.14	NA	NA
3	1.19 (0.97-1.47)	.10	1.07 (0.86-1.34)	.53	NA	NA
4	1.45 (1.16-1.82)	.001	1.18 (0.92-1.50)	.19	NA	NA

Abbreviations: AC30, at least 1 acute care use in the 30 days after starting systemic therapy; ECOG, Eastern Cooperative Oncology Group; ED, emergency department; ESAS, Edmonton Symptom Assessment System; NA, not applicable; OR, odds ratio.

^a^ Level 1 represents centers with investigational new drug programs, 2 represents centers that perform high-complexity procedures such as head and neck chemoradiotherapy, 3 represents centers with medical oncologists on site, and 4 represents satellite centers without medical oncologists on site.^30^

The PROACCT score was created using coefficients from the 3 characteristics in the final multivariable model in the development cohort (Table 2). The score ranged from 0 to 13 (Table 3). The characteristics within the score were available for 11 517 patients (94.7%) in the development cohort and 14 649 patients (92.5%) in the validation cohort. The score captured most of the discrimination of the model, with a C statistic of 0.67 (95% CI, 0.66-0.69; *P* < .001) in the development cohort (Figure, A).

**Table 3.** Prediction of Acute Care Use During Cancer Treatment Score

Characteristic	Points^a^
Age, y	
18-44	1
>75	1
Emergency department visit in past 12 mo	3
Treatment-tumor combination risk	
Low	3
Moderate	5
High	7
Very high	9

^a^ For the presence of each characteristic and summed to generate an individualized score.

**Figure.**
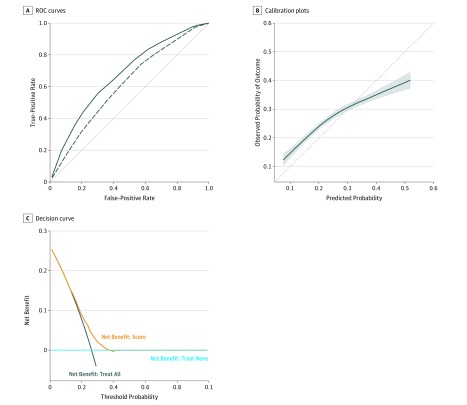
Development and Validation Cohort Curves A, Receiver operating characteristic (ROC) curves in the development (solid line) and validation (dashed line) cohorts. Derivation area under the curve (AUC) = 0.67; and validation AUC = 0.61. The dotted line is the 45° line, which corresponds to the performance of random guesses. B, Calibration plots with a locally estimated scatterplot smoothing curve and 95% CI (shaded area) in the validation cohort. The dotted line is the 45° line, which corresponds to perfect calibration. C, Decision curve in the validation cohort.

### Validation of the PROACCT Score

In the validation cohort, the PROACCT score had a C statistic of 0.61 (95% CI, 0.60-0.62; *P* < .001) (Figure, A). The risk of AC30 was significantly associated with the PROACCT score in the validation cohort (odds ratio per point increase, 1.22; 95% CI, 1.20-1.24; *P* < .001). The score was reasonably calibrated, slightly underestimating AC30 up to a score of 10, and then overestimating for scores of 12 and 13 (Figure, B and Table 4).

**Table 4.** Predicted Probability of Acute Care Use Within 30 Days of Starting Systemic Therapy by PROACCT Score and Observed Percentages in Development and Validation Cohorts

Score	Probability	Patients, No./Total No. (%)	
		Development Cohort	Validation Cohort
0	0.08	22/402 (5.5)	42/462 (9.1)
1	0.09	18/207 (8.7)	29/238 (12.2)
3	0.13	168/1261 (13.3)	290/1513 (19.2)
4	0.15	81/546 (14.8)	117/615 (19.0)
5	0.18	116/593 (19.6)	137/664 (20.6)
6	0.21	220/955 (23.0)	324/1198 (27.0)
7	0.25	146/676 (21.6)	225/800 (28.1)
8	0.29	209/732 (28.6)	306/1024 (29.9)
9	0.33	135/409 (33.0)	174/567 (30.7)
10	0.37	288/768 (37.5)	339/967 (35.1)
11	0.42	86/182 (47.3)	122/335 (36.4)
12	0.47	204/445 (45.8)	220/596 (36.9)
13	0.52	55/117 (47.0)	63/157 (40.1)

Abbreviation: PROACCT, Prediction of Acute Care Use During Cancer Treatment.

Decision curve analysis for the PROACCT score in the validation cohort is displayed in Figure, C. This decision curve demonstrates that selecting patients for an intervention using the PROACCT score had an appreciable net benefit compared with the treat all and treat none strategies for threshold probabilities of approximately 0.2 to 0.35. Therefore, selecting patients for a preventive intervention for AC30 using the PROACCT score provides a net benefit if a decision-maker is willing to apply the intervention to between 3 and 5 patients to include 1 patient who would have had an AC30.

### Sensitivity Analysis

We generated a multivariable logistic regression model with backward selection from all available characteristics, including those that might not be readily available to clinicians: Aggregated Diagnosis Groups, income quintile, rural residence, facility level, ESAS score, ECOG score, and the receipt of radiotherapy. The resulting model included the same characteristics as the PROACCT score plus rural residence, Aggregated Diagnosis Groups, and ESAS scores for drowsiness, lack of appetite, shortness of breath, anxiety, and well-being. Discrimination was only slightly improved (development cohort C statistic, 0.69). Given that this model had comparable performance but more complexity and included characteristics that may be difficult to obtain at the bedside, we used the simpler model to generate the score.

We also assessed the performance of the PROACCT score for the separate components of AC30. For ED visits that did not result in hospitalizations, the C statistic was 0.60 (95% CI, 0.59-0.61; *P* < .001) in the validation cohort. For ED visits resulting in hospitalizations or direct admissions, the C statistic was 0.59 (95% CI, 0.56-0.62; *P* < .001) in the validation cohort. We also assessed the score in palliative treatment–intent and nonpalliative treatment–intent subgroups, where the C statistics in the validation cohorts were 0.64 (95% CI, 0.63-0.65; *P* < .001) for the palliative treatment–intent cohort and 0.60 (95% CI, 0.58-0.61; *P* < .001) for the nonpalliative treatment–intent cohort.

## Discussion

In this study, we developed and validated the PROACCT score to predict AC30. The score was developed using administrative and clinical data from the population of Ontario that captured a rich set of predictive characteristics including the specific treatment regimen and patient-reported symptoms. The characteristics in the score are readily available to clinicians and recorded within most administrative databases, making the score calculable at the bedside or by electronic health records to provide personalized estimates of the risk of early use of acute care. These estimates can be used to improve the informed consent process and select patients for preventive interventions.

Our study had a large sample size with a rich set of characteristics including treatment, demographic, comorbidity, and symptom data. Although many characteristics such as patient-reported symptoms had strong univariable associations with AC30, the full model including all available characteristics had a performance similar to the simpler PROACCT score based only on cancer type, treatment regimen, ED visits, and age (C statistics in the development cohort: full model, 0.70; and simple model, 0.68). Patient-reported symptoms were of limited additional value in predicting acute care use. In the validation cohort, the PROACCT score had a C statistic of 0.61. These results suggest that characteristics observed in our data may explain only a portion of acute care use, with the remainder owing to randomness; unavailable characteristics such as laboratory test data^14^; difficult-to-measure characteristics such as patient beliefs,^42,43^ self-management skills, and behaviors^44^; or the availability of other health system supports.^5,6^

The score was reasonably calibrated in a geographically distinct validation cohort. Calibration is often underappreciated^45^ but is important for decision-making by patients, clinicians, and policy makers. For instance, a recent survey of patients with cancer found that information on the risk of acute care contributes to patient decision-making regarding chemotherapy.^3^ Moreover, the PROACCT score provided a net benefit across reasonable probability thresholds using decision curve analysis, which incorporates both discrimination and calibration.^41^ Decision curve analysis demonstrated that using the score to select patients for a proactive preventive intervention would be beneficial if a decision-maker was willing to apply the intervention to between 3 and 5 patients to include 1 patient who would have AC30. This range may include the cost-benefit ratios for many interventions such as proactive symptom monitoring using electronic or interactive smartphone applications.^46,47^

Previous studies generated models to predict hospitalizations and toxic effects of chemotherapy based on smaller samples. Brooks et al^4^ developed a logistic regression model to predict hospitalizations in a cohort of 1579 patients receiving palliative chemotherapy at a single institution, with a C statistic of 0.71 based on internal bootstrapping. In a subsequent study, Brooks et al^14^ used logistic regression to predict hospitalizations among 4240 patients with stage IV or recurrent solid tumors within 3 Kaiser Permanente regional health systems. Their model included 2 variables, albumin and sodium, and had a C statistic of 0.69 in the validation cohort.

Several other scores predict specific toxic effects of chemotherapy.^10,11,12^ For instance, Lyman et al^12^ developed a logistic regression model to predict neutropenic complications in 3760 patients from 115 centers in the United States, with a C statistic of 0.805 using a 2:1 random split for validation. To our knowledge, the PROACCT score is the first to predict both ED visits and hospitalizations in a diverse cohort of patients across all stages of disease including solid and hematologic malignant neoplasms, derived from population-based administrative data.

### Limitations

Our study should be interpreted in the context of its potential limitations. First, while we validated the score using a geographically distinct region of Ontario, prospective validation in an independent cohort would provide the strongest assessment of the score.^22^ Specifically, validation in other health care settings, including the United States, may facilitate the implementation of novel models of cancer care such as the Oncology Care Model.^8^ Second, additional characteristics that were unavailable such as laboratory test data and comprehensive geriatric assessments, which were predictive in other scores,^10,11,12,13,14^ may further refine predictions. Third, we focused on all acute care use, rather than treatment-related or preventable visits. Previous studies suggest that 25% to 75% of acute care use while undergoing systemic therapy may be treatment related^4,48^ and a similar portion may be preventable.^4,49^ Future research should develop and validate approaches to identify treatment-related and preventable acute care use from administrative data. Fourth, we used logistic regression to create a score that can be easily calculated and interpreted. Machine learning approaches may provide additional gains in predictive accuracy, although a recent review suggests that machine learning approaches seem to add limited value compared with logistic regression for clinical prediction models.^50^

## Conclusions

The PROACCT score predicts the risk of acute care use after the initiation of systemic therapy for cancer. The score quantifies an important risk of systemic therapy, which can improve the informed consent process. Future research should determine how the score affects clinical decision-making and how the score can be used to guide preventive interventions to reduce morbidity and costs.

## Notes

Supplement. **eTable.** Classification of Systemic Therapies by the Risk of an Emergency Department Visit or Hospitalization Within Thirty Days of Initiation of Treatment
