# Supplementary material for: Development and Validation of a Score to Predict Acute Care Use After Initiation of Systemic Therapy for Cancer
Source: JAMA Netw Open. 2019 Oct 9;2(10):e1912823. doi: 10.1001/jamanetworkopen.2019.12823 (PMC6802230; doi:10.1001/jamanetworkopen.2019.12823)
Supplement: Supplement. — eTable. Classification of Systemic Therapies by the Risk of an Emergency Department Visit or Hospitalization Within Thirty Days of Initiation of Treatment [file jamanetwopen-2-e1912823-s001.pdf]

Supplementary Online Content

Grant RC, Moineddin R, Yao Z, Powis M, Kukreti V, Krzyzanowska MK. Development and validation of a score to predict acute care use after initiation of systemic therapy for cancer. *JAMA Netw Open.* 2019;2(10):e1912823. doi:10.1001/jamanetworkopen.2019.12823

eTable. Classification of Systemic Therapies by the Risk of an Emergency Department Visit or Hospitalization Within Thirty Days of Initiation of Treatment

This supplementary material has been provided by the authors to give readers additional information about their work.

© 2019 Grant RC et al. *JAMA Network Open.*

eTable. Classification of Systemic Therapies by the Risk of an Emergency Department Visit or Hospitalization Within Thirty Days of Initiation of Treatment

Cancer	Regimen	Patients in the development cohort	Patients with AC30 in the development cohort	Mean acute care visits in 30 days in the development cohort	Quintile	Patients in the validation cohort	Patients with AC30 in the validation cohort
Anus	FUMTMC(RT)	65	19	0.43	4	96	19
Bladder	CISP(RT-W)	22	5	0.27	2	23	7
Bladder	CISPGEMC	85	38	0.59	5	171	56
Bladder	CRBP(RT)	23	5	0.26	2	1	1
Bladder	CRBPGEMC	37	16	0.65	5	62	25
Bladder	FUMTMC(RT)	16	4	0.25	2	8	4
Bladder	MVAC(HD)	21	3	0.14	1	8	3
Brain	LOMU	22	9	0.59	5	44	11
Brain	TMZL	178	33	0.26	2	262	59
Brain	TMZL(RT)-TMZL	202	35	0.24	2	124	21
Breast	AC	98	24	0.38	3	116	28
Breast	AC-DOCE	45	11	0.29	2	19	8
Breast	AC-PACL	53	12	0.25	2	27	11
Breast	AC-PACL(DD)	534	107	0.26	2	414	91
Breast	AC-PACL(DD)+TRAS	17	2	0.18	1	31	3
Breast	AC-PACL(W)	54	11	0.22	2	92	36
Breast	AC-PACL(W)+TRAS	22	4	0.23	2	23	7
Breast	CAPE	200	43	0.28	2	201	37
Breast	CAPELAPA	10	0	0.00	1	9	2
Breast	CISPGEMC(W)	15	5	0.33	3	39	9
Breast	CRBPDOCETRAS	21	3	0.14	1	35	11
Breast	CYCLDOCE	270	73	0.35	3	412	125
Breast	CYCLDOCE+TRAS	41	6	0.22	2	66	21
Breast	DOCE	39	21	0.69	5	32	10

© 2019 Grant RC et al. *JAMA Network Open.*

Cancer	Regimen	Patients in the development cohort	Patients with AC30 in the development cohort	Mean acute care visits in 30 days in the development cohort	Quintile	Patients in the validation cohort	Patients with AC30 in the validation cohort
Breast	DOCE+PERT+TRAS	15	7	0.73	5	22	5
Breast	DOXO(W)	29	6	0.21	2	19	6
Breast	ERIB	36	14	0.47	4	48	18
Breast	EVEREXEM	44	6	0.18	1	62	17
Breast	FEC-D	183	39	0.27	2	636	155
Breast	FEC-D+TRAS	65	13	0.22	2	155	45
Breast	FEC100	153	44	0.42	3	144	35
Breast	FLVS	84	7	0.10	1	103	11
Breast	KADC	41	7	0.17	1	42	10
Breast	NPAC	14	5	0.36	3	7	1
Breast	PACL(W)	140	39	0.35	3	220	50
Breast	PACL(W)+PERT+TRAS	13	1	0.15	1	34	11
Breast	PACL(W)+TRAS	24	5	0.25	2	52	5
Breast	PACL+PERT+TRAS	17	2	0.12	1	25	5
Breast	PERT+TRAS	41	10	0.34	3	40	8
Breast	TRAS	155	29	0.23	2	193	21
Breast	VINO	25	4	0.16	1	57	11
Cervix	CISP(RT-W)	85	21	0.38	3	120	31
Cervix	CISPPACL	10	1	0.20	2	6	3
Cervix	CRBPPACL	14	6	0.57	5	27	11
Chronic Lymphocytic Leukemia	BEND	46	13	0.41	3	43	17
Chronic Lymphocytic Leukemia	BEND+RITU	20	7	0.40	3	20	7
Chronic Lymphocytic Leukemia	CHLO	34	6	0.24	2	26	4
Chronic Lymphocytic Leukemia	FC(PO)+R	22	5	0.32	3	11	3
Chronic Lymphocytic Leukemia	FC+R	48	12	0.27	2	84	19
Chronic Lymphocytic Leukemia	FLUD(PO)+R	10	1	0.10	1	6	3
Chronic Lymphocytic Leukemia	IBRU	21	2	0.10	1	41	8

© 2019 Grant RC et al. *JAMA Network Open.*

Cancer	Regimen	Patients in the development cohort	Patients with AC30 in the development cohort	Mean acute care visits in 30 days in the development cohort	Quintile	Patients in the validation cohort	Patients with AC30 in the validation cohort
Colorectal	CAPE	473	53	0.17	1	437	65
Colorectal	CAPE(RT)	158	24	0.20	2	274	29
Colorectal	FOLFIRI	78	18	0.31	3	128	39
Colorectal	FOLFIRI+BEVA	361	87	0.31	3	510	145
Colorectal	FU(CIV-RT)	27	1	0.04	1	52	8
Colorectal	IRIN	17	7	0.65	5	7	4
Colorectal	IRIN(Q2W)+CETU	26	4	0.15	1	12	2
Colorectal	MFOLFOX6	564	107	0.26	2	845	166
Colorectal	MFOLFOX6+BEVA	84	21	0.37	3	62	18
Colorectal	PNTM	29	5	0.24	2	51	14
Colorectal	REGO	20	3	0.20	2	25	6
Colorectal	XELOX	17	4	0.41	3	19	10
Colorectal	XELOX+BEVA	10	5	0.50	4	4	1
Sarcoma	DOCEGEMC	11	9	1.00	5	7	3
Sarcoma	DOXO	12	6	1.00	5	37	13
Gastroesophageal	CAPECISP+TRAS	19	9	0.63	5	18	6
Gastroesophageal	CISPFU	16	4	0.38	3	40	16
Gastroesophageal	CISPFU+TRAS	18	9	0.56	5	14	6
Gastroesophageal	CRBPPACL(RT)	78	15	0.28	2	126	32
Gastroesophageal	ECARBOF	13	5	0.46	4	6	3
Gastroesophageal	ECF	124	53	0.63	5	173	46
Gastroesophageal	ECX	79	27	0.49	4	83	28
Gastroesophageal	FULCVR(RT-GAST)	29	7	0.31	3	26	6
Gastroesophageal	PACL(W)	16	2	0.13	1	26	7
Head and Neck	CETU(RT)	11	3	0.36	3	38	19
Head and Neck	CISP(RT-W)	25	9	0.44	4	36	13
Head and Neck	CISP(RT)	150	49	0.43	4	171	66

© 2019 Grant RC et al. *JAMA Network Open.*

Cancer	Regimen	Patients in the development cohort	Patients with AC30 in the development cohort	Mean acute care visits in 30 days in the development cohort	Quintile	Patients in the validation cohort	Patients with AC30 in the validation cohort
Head and Neck	CISPFU	21	7	0.43	4	8	4
Head and Neck	CRBPFU(RT)	22	12	0.82	5	2	1
Head and Neck	CRBPPACL	20	10	0.75	5	26	12
Hepatobiliary	CISPGEMC	34	19	0.91	5	39	20
Hepatobiliary	CISPGEMC(W)	37	14	0.59	5	63	23
Hepatobiliary	GEMC	26	5	0.46	4	32	8
Hepatobiliary	SORA	19	2	0.11	1	43	12
Hodgkins Lymphoma	ABVD	123	31	0.41	3	140	41
Hodgkins Lymphoma	GDP	16	6	0.50	4	19	3
Intermediate Grade Lymphoma	CHOP	24	10	0.67	5	28	12
Intermediate Grade Lymphoma	CHOP+R	273	95	0.52	4	415	157
Intermediate Grade Lymphoma	GDP	23	9	0.48	4	44	22
Low Grade Lymphoma	BEND	10	3	0.40	3	8	4
Low Grade Lymphoma	BEND+RITU	168	37	0.30	3	239	57
Low Grade Lymphoma	CHOP+R	30	13	0.60	5	17	5
Low Grade Lymphoma	CVP+R	19	1	0.11	1	14	6
Low Grade Lymphoma	RITU	12	3	0.33	3	19	4
Low Grade Lymphoma	RITU(MNT)	238	5	0.02	1	297	23
Myelodysplastic syndrome	AZCT	63	19	0.44	4	69	29
Myelodysplastic syndrome	LENA	10	3	0.40	3	12	1
Melanoma	CRBPPACL	11	5	0.45	4	20	4
Melanoma	DABR	24	5	0.21	2	19	5
Melanoma	DCRB	42	16	0.48	4	53	12
Melanoma	IFNA(IND-MNT(SC))	57	6	0.12	1	49	8
Melanoma	IPIL	60	15	0.33	3	66	19
Melanoma	TMZL	12	5	0.58	5	0	0
Melanoma	VEMU	24	4	0.17	1	14	2

© 2019 Grant RC et al. *JAMA Network Open.*

Cancer	Regimen	Patients in the development cohort	Patients with AC30 in the development cohort	Mean acute care visits in 30 days in the development cohort	Quintile	Patients in the validation cohort	Patients with AC30 in the validation cohort
Myeloma	BMP	93	27	0.35	3	188	62
Myeloma	BORT	84	25	0.39	3	50	12
Myeloma	CYBORD	143	39	0.44	4	187	64
Myeloma	CYCL	19	3	0.16	1	59	20
Myeloma	CYCL(PO)	22	8	0.50	4	11	6
Myeloma	DEXALENA	170	22	0.18	1	132	28
Myeloma	DEXAPOMA	16	2	0.13	1	11	5
Myeloma	LENA	79	11	0.18	1	80	7
Myeloma	MELPPRED	16	3	0.25	2	18	3
Neuroendocrine tumours	CISPETOP(3D)	15	7	0.53	5	18	5
Neuroendocrine tumours	OCTR	104	20	0.22	2	82	10
Nonsmall cell lung cancer	AFAT	17	5	0.35	3	37	9
Nonsmall cell lung cancer	CISPETOP(3D)	38	13	0.45	4	47	15
Nonsmall cell lung cancer	CISPETOP(RT)	123	30	0.30	3	137	38
Nonsmall cell lung cancer	CISPGEMC	72	26	0.50	4	62	28
Nonsmall cell lung cancer	CISPPEME	97	35	0.59	5	226	75
Nonsmall cell lung cancer	CISPVINO	74	17	0.31	3	162	37
Nonsmall cell lung cancer	CISPVINO(W)	61	18	0.43	4	45	17
Nonsmall cell lung cancer	CISPVNBL	38	17	0.53	5	1	0
Nonsmall cell lung cancer	CRBPETOP	48	18	0.65	5	25	13
Nonsmall cell lung cancer	CRBPETOP(RT)	14	5	0.50	4	23	6
Nonsmall cell lung cancer	CRBPGEMC	152	61	0.60	5	107	37
Nonsmall cell lung cancer	CRBPPACL	54	10	0.20	2	60	23
Nonsmall cell lung cancer	CRBPPEME	141	50	0.49	4	192	53
Nonsmall cell lung cancer	CRBPVINO	15	5	0.53	5	54	15
Nonsmall cell lung cancer	CRBPVNBL	41	14	0.44	4	0	0
Nonsmall cell lung cancer	CRIZ	20	7	0.65	5	20	2

© 2019 Grant RC et al. *JAMA Network Open.*

Cancer	Regimen	Patients in the development cohort	Patients with AC30 in the development cohort	Mean acute care visits in 30 days in the development cohort	Quintile	Patients in the validation cohort	Patients with AC30 in the validation cohort
Nonsmall cell lung cancer	DOCE	41	25	0.78	5	49	23
Nonsmall cell lung cancer	ERLO	71	20	0.34	3	69	18
Nonsmall cell lung cancer	GEFI	150	22	0.19	1	183	23
Nonsmall cell lung cancer	PEME	99	22	0.25	2	164	48
Nonsmall cell lung cancer	VINO	10	3	0.40	3	30	14
Ovarian	CRBP	55	19	0.47	4	117	31
Ovarian	CRBPGEMC	24	5	0.29	2	9	1
Ovarian	CRBPPACL	279	86	0.46	4	369	105
Ovarian	CRBPPACL(IP)	21	7	0.48	4	19	6
Ovarian	GEMC	17	4	0.41	3	24	8
Ovarian	PACL(W)	23	7	0.57	5	45	8
Ovarian	PGLDX	56	18	0.48	4	66	27
Pancreas	FOLFIRINOX	136	52	0.57	5	184	80
Pancreas	FU(CIV-RT)	12	1	0.08	1	11	2
Pancreas	GEMC	158	47	0.46	4	209	69
Pancreas	GEMCNPAC(W)	33	17	0.79	5	80	36
Prostate	ABIRPRED	249	33	0.16	1	269	56
Prostate	DOCE	91	32	0.54	5	15	9
Prostate	DOCEPRED	98	37	0.46	4	199	50
Prostate	ENZL	136	18	0.16	1	181	27
Renal	EVER	15	2	0.13	1	20	5
Renal	PAZO	32	10	0.50	4	20	7
Renal	SUNI	76	17	0.28	2	131	29
Small cell lung cancer	CISPETOP(3D)	103	34	0.44	4	140	51
Small cell lung cancer	CRBPETOP	116	40	0.48	4	156	61
Small cell lung cancer	ETOP(PO)	12	3	0.25	2	2	0
Small bowel appendix	MFOLFOX6	25	4	0.20	2	19	4

© 2019 Grant RC et al. *JAMA Network Open.*

Cancer	Regimen	Patients in the development cohort	Patients with AC30 in the development cohort	Mean acute care visits in 30 days in the development cohort	Quintile	Patients in the validation cohort	Patients with AC30 in the validation cohort
Unknow primary site	CISPETOP(3D)	10	5	0.50	4	4	0
Unknow primary site	CRBPPACL	12	9	1.17	5	27	8
Uterus	CRBP	33	5	0.15	1	28	6
Uterus	CRBPPACL	185	43	0.29	3	224	54
Uterus	DOXO	11	5	0.64	5	12	3

AC30: Acute care use within 30 days.

© 2019 Grant RC et al. *JAMA Network Open.*
